# Predictions for optimal mitigation of paracrine inhibitory signalling in haemopoietic stem cell cultures

**DOI:** 10.1186/s13287-015-0048-7

**Published:** 2015-04-16

**Authors:** Joseph D Berry, Pankaj Godara, Petar Liovic, David N Haylock

**Affiliations:** Commonwealth Scientific and Industrial Research Organisation Mineral Resources (CSIRO), Clayton, Victoria 3168 Australia; Commonwealth Scientific and Industrial Research Organisation (CSIRO) Manufacturing, Clayton, Victoria 3168 Australia

## Abstract

**Introduction:**

Recent studies in the literature have highlighted the critical role played by cell signalling in determining haemopoietic stem cell (HSC) fate within *ex vivo* culture systems. Stimulatory signals can enhance proliferation and promote differentiation, whilst inhibitory signals can significantly limit culture output.

**Methods:**

Numerical models of various mitigation strategies are presented and applied to determine effectiveness of these strategies toward mitigation of paracrine inhibitory signalling inherent in these culture systems. The strategies assessed include mixing, media-exchange, fed-batch and perfusion.

**Results:**

The models predict that significant spatial concentration gradients exist in typical cell cultures, with important consequences for subsequent cell expansion. Media exchange is shown to be the most effective mitigation strategy, but remains labour intensive and difficult to scale-up for large culture systems. The fed-batch strategy is only effective at very small Peclet number, and its effect is diminished as the cell culture volume grows. Conversely, mixing is effective at high Peclet number, and ineffective at low Peclet number. The models predict that cell expansion in fed-batch cultures becomes independent of increasing dilution rate, consistent with experimental results previously reported in the literature. In contrast, the models predict that increasing the flow rate in perfused cultures will lead to increased cell expansion, indicating the suitability of perfusion for use as an automated, tunable strategy. The effect of initial cell seeding density is also investigated, with the model showing that perfusion outperforms dilution for all densities considered.

**Conclusions:**

The models predict that the impact of inhibitory signalling in HSC cultures can be mitigated against using media manipulation strategies, with the optimal strategy dependent upon the protein diffusion time-scale relative to the media manipulation time-scale. The key messages from this study can be applied to any complex cell culture scenario where cell-cell interactions and paracrine signalling networks impact upon cell fate and cell expansion.

**Electronic supplementary material:**

The online version of this article (doi:10.1186/s13287-015-0048-7) contains supplementary material, which is available to authorized users.

## Introduction

Haemopoietic stem cells (HSCs) ultimately give rise to all blood cells, and as a consequence hold great promise for *ex vivo* production of mature blood cells for blood transfusion. However, the quantity of HSCs able to be harvested from patients is insufficient to generate the enormous numbers of cells required for clinical use. Hence, there is a critical need to increase the number of HSCs for mature cell biomanufacture [1]. A common approach used to expand HSCs is to culture them under static conditions in fully defined media without serum but with supplementation of early acting synergistic factors that promote HSC survival and proliferation [2-6]. Protocols for the expansion of HSCs are usually formulated to ensure that sufficient growth factors are provided in the initial cell culture medium for the duration of the culture period. More sophisticated *ex vivo* culture systems utilise various feeding strategies to provide sustained levels of the key haemopoietic growth factors required for maximal cell production at the end of the culture period.

The fate, proliferation and differentiation of HSCs within *ex vivo* culture systems are ultimately determined by the interplay between the intrinsic properties of the HSCs and a multitude of extrinsic signals that collectively influence growth. Simplistically, extrinsic cues can be considered as either stimulatory or inhibitory and the relative magnitudes of these competing influences will determine HSC response. Until recently, HSC expansion strategies have focussed mainly on what combination and concentration of stimulatory regulators need to be provided to ensure optimal cell proliferation and self-renewal decisions. However, recent studies from Zandstra and colleagues have highlighted the influence of combinations of cell-synthesised inhibitory proteins present at subthreshold levels that significantly limit expansion of HSC and their immediate progeny [7-10]. These negative feedback regulatory loops are important in HSC cultures, especially those where cells are seeded at high density, resulting in minimal distance between precursor cells and/or their nascent progeny.

Bioreactor systems for HSC growth and expansion should be designed to provide adequate amounts of stimulatory factors and cytokines, glucose and other essential metabolites to promote the survival and division of cells, but also remove or mitigate the effect of cell-synthesised inhibitors. Approaches for mitigating the effect of these inhibitors include but are not limited to: mixing and redistribution of cells and media; removal of inhibitory factors by media exchange; removal of inhibitory factors by continuous media perfusion; dilution of inhibitory factors by continuous media addition (fed-batch culture); sequestration of inhibitory factors; and targeted molecular negation of specific inhibitory factors.

Models that predict growth of HSC cultures under different mitigation strategies enable rapid and efficient identification of optimal operating conditions for *ex vivo* culture systems, eliminating the need for expensive and time-consuming device prototyping. Kirouac et al. formulated a HSC growth model to describe regulation of HSC populations through intercellular communication caused by cell-secreted factors [9]. This was initially applied to static cultures (with and without media exchange), and was extended by Csaszar and colleagues to include perfused and fed-batch cultures [8]. The model is formulated under the assumption that these secreted factors are instantaneously redistributed throughout the cell culture. This assumption implies that the proteins secreted by cells are effectively massless, and concentration gradients are not present in typical cell cultures.

Herein we describe a mathematical approach for considering the impact of inhibitory proteins on HSC growth, based on consideration of their molecular mass and size. We use analytical and numerical models to predict optimal strategies for mitigating the effects of inhibitory signalling on the expansion of HSC populations.

## Materials and methods

The equations and models used in this study are presented in Additional file 1. To determine the evolution of the secreted factor *ϕ* in static, mixed, fed-batch and media-exchange cell cultures under the assumption of fixed and uniform cell density, the governing equations were discretised and solved using the FiPy partial differential equation solver [11]. The domain was split into 100 equal elements, and a time step of 300 seconds was used. When the assumption of fixed cell number was relaxed, the Gear method was used to solve the governing equations for these cultures as an initial value problem, available as a standard numerical library in Python (http://www.python.org). The method of lines was used to discretise the transient diffusion equation spatially, allowing it to be solved using the Gear method. The domain was split into 200 elements to accommodate the growth of the domain in the Eulerian frame.

The governing equations for the perfusion culture were solved using ANSYS-CFX (ANSYS, Canonsburg, PA, USA), with a constant time step of 400 seconds. The symmetry of the geometry was exploited to reduce computational expense. The mesh size used was approximately 27,000 nodes, and the mesh was graded to ensure that large spatial gradients of *ϕ* near the bottom of the cell culture were sufficiently resolved. Tests were performed to ensure that the mesh resolution was of adequate size to provide accurate results. Tests were also performed to ensure that, with *Q* = 0, the growth model for both the perfused and fed-batch cultures gave the same result at the static culture limit.

## Results

**Figure 1:**
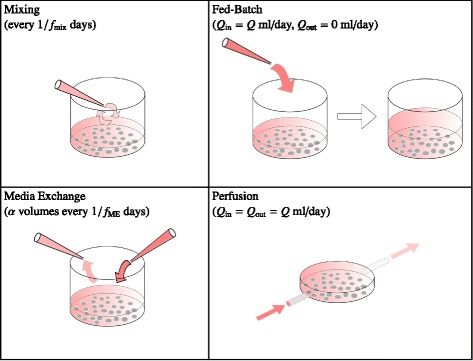
Mitigation strategies for minimising the effects of paracrine inhibitory signalling. Mixing: cell culture medium is drawn up and down repeatedly. The mixing frequency *f*_mix_ determines how often mixing occurs. The volume of the culture remains constant. Media-exchange: a fraction *α* of the total cell culture volume is replaced by fresh media after every time interval 1/*f*_ME_, where *f*_ME_ is the media-exchange frequency. Again, the volume of the culture remains constant. Fed-batch: fresh medium is continuously added to the culture at a constant rate of *Q* ml/day. Medium is not removed from the culture, and hence the culture volume increases over time. Perfusion: fresh medium is continually added to the culture system, and spent medium is continually removed from the culture system, both at a constant rate of *Q* ml/day. The cell culture volume remains constant over time.

In this study, models of different strategies for mitigating paracrine inhibitory signalling on the expansion of HSC populations during culture are considered. These include mixing, media-exchange, fed-batch and perfusion strategies (Figure 1). The mixing strategy consists of manipulating existing cell media in order to redistribute inhibitory proteins evenly throughout the cell culture volume. The mixing frequency *f*_mix_ determines how often mixing occurs. For example, if *f*_mix_ = 1 then mixing occurs daily. For media-exchange strategies a fraction *α* of the total cell culture volume is replaced by new media after every time interval 1/*f*_ME_, where *f*_ME_ is the media-exchange frequency. Both mixing and media-exchange strategies can be considered discrete strategies, as media manipulation only occurs at particular instants in time during cell culture.

In contrast, fed-batch and perfusion cultures as considered in this study are both continuous strategies. For fed-batch cultures, medium is assumed to be continuously added at a constant rate of *Q* ml/day. No medium is removed from the culture, and thus the inhibitory protein concentration is diluted as the culture volume increases. In practice, it is difficult for dilution to be a continuous process: Csaszar and colleagues performed experiments using a semi-continuous dilution regime with 30 minute dilution cycles [8]. In our models we assume that dilution acts continuously, which gives an upper limit for the performance of fed-batch systems. Further, the models here assume that the action of dilution imparts no mixing on the media already present, which is a reasonable assumption if medium is added at very low flow rates (see Additional files 2 and 3). In a perfused culture, fresh medium is continually delivered to the culture system, and spent medium is continually removed, at the constant rate of *Q* ml/day, such that the cell culture volume remains constant over time.

We are assuming in the numerical models that the cell redistribution due to the media manipulations has a negligible effect on the subsequent growth of the cell population. This is a reasonable assumption for both the perfusion and dilution strategies, because the shear stresses exerted on the cells for the flow rates considered are extremely low. However, cell redistribution of the cells is a natural consequence of the mixing and media exchange strategies. The effect of this redistribution is unknown, and very difficult to quantify because it is an inherent part of practical media manipulation. The action of cell redistribution may have a positive effect on cell expansion because it exposes cells to new neighbours, but it may also be deleterious because the close cell contact necessary for effective juxtacrine and paracrine signalling will be periodically disrupted. We also assume here that laboratory incubator vibrations have negligible effect on mixing in the cell cultures, because they are ill-defined and can be easily negated with the use of vibration damping systems.

Two types of fed-batch devices are considered in this study: a circular well with the same footprint as a single well on a standard tissue culture 24-well plate, and a 12 ml culture bag. Cells are considered to reside on the bottom surface of each device due to the action of gravity. The surface area of the well is 2 cm^2^, and the surface area of the culture bag is 30 cm^2^. For the perfused culture scenarios considered, the inlet and outlet pipes are located halfway up the well, and the top surface of the culture is considered to be a solid wall. If *Q* = 0 ml/day for either the fed-batch or the perfused culture, the system reduces to a static culture. The well culture device is assumed to be manufactured from thin polydimethylsiloxane, which allows oxygen to permeate through the walls of the device [12-14], therefore placing no practical limitation owing to oxygen diffusion considerations on the height of the media within the device.

To first assess the effectiveness of various strategies to mitigate inhibitory signalling, the assumption of a fixed cell population is made. Under this assumption, if one strategy always gives a lower inhibitory concentration where the cells reside than another, this will lead to a greater cell expansion when applied to a population of secreting cells. However, if one strategy outperforms another for some stages of the cell culture period, but is worse for other stages, the determination of which strategy is better will depend upon the dynamics of the particular cell population undergoing expansion. To determine the impact of cell population, the fixed-cell assumption is relaxed and the growth model of Kirouac and colleagues [9] and Csaszar and colleagues [8] is extended and applied to assess the cell expansion predictions of each mitigation strategy. We use transforming growth factor beta (TGF-β) as the model inhibitory signal emitted by the HSC population because it is known to inhibit haemopoietic progenitors [15]. Table 1 presents estimates of the diffusivity of TGF-β, along with other known inhibitory proteins relevant to HSC cultures. Whilst the secretion rate of the model signal is assumed to be the same as the estimate given by Kirouac and colleagues [16] (Table 2), experimentally measured TGF-β concentrations in HSC cultures indicate the actual secretion rate to be higher [8,10]. This is consistent with the solitary cell model developed by Francis and Palsson [17], who show that for effective intercellular communication to occur the ratio of the diffusive time scale to the secretion time scale (defined as α) needs to be O(1) or greater. The secretion rates reported by Kirouac and colleagues [9] give α ~ 5 × 10^−3^. Thus it is likely that actual secretion rates are at least two orders of magnitude higher.

**Table 1:** **Physical properties of known haemopoietic stem cell inhibitory factors**

**Inhibitory factor**	**Molecular weight (kDa)**	**Diffusivity (m** ^**2**^ **/second)**
TGF-β	26	6.43 × 10^−11^
TNFSF9	23.8	6.76 × 10^−11^
MIP-1α	8	1.23 × 10^−10^
MIP-1β	8	1.23 × 10^−10^
IP-10	9	1.16 × 10^−10^
NAP-2	8	1.23 × 10^−10^
SPARC	40	5.07 × 10^−11^
PDGF-CC	13.4	9.28 × 10^−11^

The diffusivity is calculated using the formula *D* = 1.72 × 10^−8^(MW)^−0.552^ [32,33]. IP, interferon gamma induced protein; MIP, macrophage inhibitory protein; MW, molecular weight; NAP, neutrophil activating protein; PDGF, platelet-derived growth factor; SPARC, secreted protein, acidic and rich in cysteine; TGF-β, transforming growth factor beta; TNF, tumour necrosis factor.

**Table 2:** **Growth model parameters** [9,29]

**Parameter**	**Symbol**	**Value**	**Units**
Maximum proliferation rate of Lin^−^ cells	*u*_*max*_	6.26	1/day
Maximum proliferation rate of Lin^+^ cells	*u*_+_	0.204	1/day
Maximal proliferation compartment	*n*_*max*_	5.32	–
Proliferative decay term	*D*_*gr*_	3.38	–
Self-renewal probability	*f*_*max*_	0.634	–
Self-renewal decay term	*D*_*sr*_	1.96	–
First SF1 Hill coefficient	*k*_1_	0.614	–
Second SF1 Hill coefficient	*k*_s_	1.08	–
SF2 Hill coefficient	*k*_2_	0.555	–
SF3 Hill coefficient	*k*_3_	0.625	–
SF4 Hill coefficient	*k*_4_	0.533	–
SF1 concentration inducing 50% maximal SF2 secretion	*L*_*s*_	0.915	pg/ml
Cell cycle Hill coefficient	*k*_*t*_	5	–
Time for 50% of cells to enter cycle	*τ*_*D*_	2	Days
SF1 secretion rate	*r*_1_	1.94 × 10^−6^	pg/cell/day
SF2 secretion rate	*r*_2_	2.40 × 10^−6^	pg/cell/day
SF3 secretion rate	*r*_3_	4.88 × 10^−7^	pg/cell/day
SF4 secretion rate	*r*_4_	4.34 × 10^−7^	pg/cell/day

### Fed-batch strategy is preferable for mitigating inhibitory signalling if diffusion is not considered

Csaszar and colleagues formulated a model of fed-batch and perfusion-based cultures under the assumption that inhibitory factors instantaneously redistribute throughout the cell culture volume [8]. When this assumption is made, the rate at which the concentration of inhibitory signals within the culture changes can be determined by

1 $$ \frac{d\phi (t)}{dt}=\frac{r_{\phi }X(t)-Q\phi }{V_0}, $$

2 $$ \frac{d\phi (t)}{dt}=\frac{r_{\phi }X(t)-Q\phi }{V_0+Qt}, $$

for perfused and fed-batch cultures respectively. Here *ϕ*(*t*) is the concentration of inhibitory protein at time *t*, *X*(*t*) is the number of secreting cells at time *t*, *r*_*ϕ*_ is the cell secretion rate of inhibitory protein, *Q* is the flow rate and *V*_0_ is the initial culture volume.

**Figure 2:**
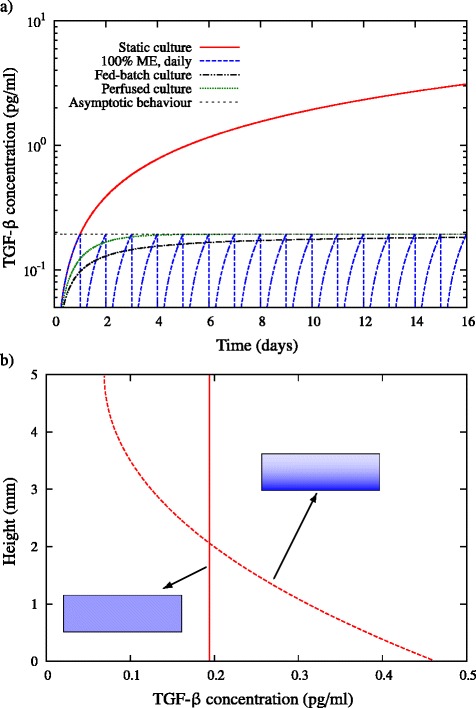
Assuming instantaneous redistribution of signalling shows that the fed-batch strategy is the most effective, but this assumption is not valid for typical culture systems. **(a)** Variation of transforming growth factor beta (TGF-β) concentration in a 24-well-plate well with time under the assumption that secreted TGF-β is instantaneously distributed throughout the cell-culture volume. Shown are the analytical solutions to the governing equations defined in Equations (1) and (2). **(b)** Spatial distribution of secreted TGF-β concentration after 1 day of static culture assuming finite diffusivity of *D* = 6.5 × 10^−11^ m^2^/second (dashed line, numerical solution of Equations (10) and (11) in Additional file 1) and infinite diffusivity (solid line, analytical solution of Equation (1) or (2) with *Q* = 0). Colour contours show a graphical representation of the concentration variation within the well at day 1 for each case. (a), (b) Cell population assumed constant at *X* = 10^5^, and the secretion rate of TGF-β is *r*_*ϕ*_ = 1.94 × 10^−6^ pg/cell/day.

The static culture case can be modelled by setting the flow rate *Q* = 0 ml/day in either equation. Under the assumption that the number of secreting cells is constant, Equations (1) and (2) can be solved analytically. These analytical solutions are shown in Figure 2a for the fed-batch, perfusion and daily media-exchange strategies for a culture of 10^5^ cells secreting TGF-β at a fixed rate of *r*_*ϕ*_ = 1.94 × 10^−6^ pg/cell/day^a^ [9], along with the solution for a static culture. The concentration of inhibitory protein in a static culture increases with time; however, the fed-batch and perfused cultures approach a steady-state value of *ϕ* = *r*_*ϕ*_*X*/*Q*, which for this particular choice of parameters is equal to 0.194 pg/ml. The maximum concentration of the media-exchange strategy also approaches this value. The fed-batch culture approaches steady-state slower than the perfused culture, and as a consequence always has the lowest relative concentration of inhibitory signal. When media exchange is considered, the peak concentration reached is always higher than that of the diluted culture. Csaszar and colleagues showed that the small increase in effectiveness of the fed-batch culture in comparison with the other strategies leads to a large increase in cell expansion [8]. Therefore, if the effects of diffusion are not considered, the fed-batch strategy is preferable for mitigating the effects of inhibitory signalling. However, in reality, inhibitory proteins have defined mass and hence diffusivity and may be unevenly distributed throughout the culture media. We have thus formulated a model that takes into account finite diffusivity to re-evaluate the effectiveness of each mitigation strategy (see Additional file 1).

### Considering the effects of diffusion gives rise to significant spatial concentration gradients

To test the validity of the instantaneous redistribution assumption, we can evaluate the importance of this by considering the inhibitory protein TGF-β, with a molecular weight of 26 kDa corresponding to a diffusivity of 6.5 × 10^−11^ m^2^/second. The height of 1 ml liquid in a 24-well-plate well is approximately 5 mm, giving a characteristic time for diffusion of *τ*_*D*_ = *L*^2^/*D* ~ 4.5 days. This time is significant in comparison with the cell culture time scale of *τ*_culture_ = 8 to 16 days. Therefore, significant concentration gradients will be present in typical cell cultures, and diffusive transport needs to be accounted for in growth models in order to accurately assess the effectiveness of mitigation strategies.

By taking into consideration the protein diffusivity we are able to predict the concentration of protein at any distance from the cellular source. To illustrate this, Figure 2b shows the concentration profile of TGF-β after 1 day in a 24-well-plate well geometry for a culture of 10^5^ cells secreting TGF-β at a fixed rate of *r*_*ϕ*_ = 1.94 × 10^−6^ pg/cell/day. The concentration of TGF-β at the bottom of the well is approximately 0.45 pg/ml, which is double the value predicted by the instantaneous redistribution model. Including the effects of diffusion thus gives rise to significant spatial concentration gradients, which need to be considered when comparing strategies to mitigate the effects of different inhibitory proteins.

### Assuming instantaneous redistribution of inhibitory signals overpredicts the effectiveness of fed-batch cultures

In fed-batch cultures, the cell culture volume is a function of time, and the characteristic dilution time scale can be shown to vary as *τ*_dilute_ ∝ 1/*Q*. If the flow rate is very low, the dilution time scale is much larger than the cell culture period, and the limiting case in this instance is a static culture. Conversely, if the flow rate is very large, the dilution time scale is much less than the cell culture period, and the media height can be considered to be infinitely large. For the large flow rate case, assuming that the dilution occurs at the top of the cell culture, an analytical solution to the transient diffusion equation (Equation (3) in Additional file 1) exists [18]. The analytical solution for concentration distribution in the case of constant flux into a semi-infinite medium is given as

3 $$ \phi \left(y,t\right)=2{r}_{\phi}\sqrt{\frac{t}{\pi D}} \exp \left(\frac{-{y}^2}{4Dt}\right)-\frac{r_{\phi }y}{D}\mathrm{erfc}\left(\frac{y}{2\sqrt{Dt}}\right), $$

where *D* is the diffusivity of the inhibitory protein, *y* is the distance above the cells, *t* is the cell culture time and erfc is the complementary error function. As we are interested in the concentration of the inhibitory signal exposed to the cells, we can set *y* = 0 to obtain

4 $$ {\phi}_{cell}(t) = 2{r}_{\phi}\sqrt{\frac{t}{\pi D}.} $$

The concentration of inhibitory protein at the bottom of a cell culture will thus increase with culture time as *ϕ*_cell_ ∝ *t*^1/2^.

**Figure 3:**
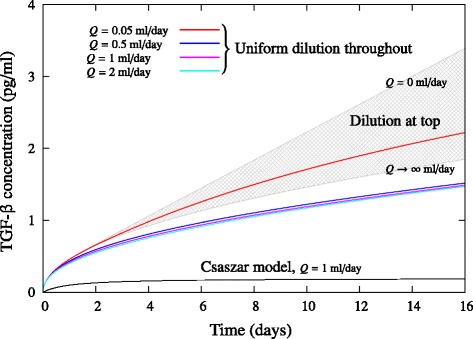
Assuming instantaneous redistribution of inhibitory signals overpredicts the effectiveness of fed-batch cultures. Effect of flow rate *Q* on the concentration of transforming growth factor beta (TGF-β) at the bottom of a 24-well-plate well of initial media height *h*_0_ = 5 mm (corresponding to 1 ml culture). Shaded grey area shows the effect of continuous dilution into the top of the culture (Equation (4)), and coloured lines show the effect of uniform dilution throughout the culture (numerical solution of Equations (10) and (11) in Additional file 1). Solid black line represents the model of Csaszar and colleagues [8], given by the analytical solution of Equation (1), whereby spatial concentration gradients are ignored (*D* → ∞). The diffusivity is *D* = 6.5 × 10^−11^ m^2^/second, the cell population is assumed constant at *X* = 10^5^, and the secretion rate is *r*_*ϕ*_ = 1.94 × 10^−6^ pg/cell/day.

The two limiting cases are shown in Figure 3, for a culture of 10^5^ cells in a well from a 24-well plate secreting TGF-β at a fixed rate of *r*_*ϕ*_ = 1.94 × 10^−6^ pg/cell/day. The upper grey curve corresponds to the static culture limit (*Q* = 0), solved numerically, and the lower grey curve corresponds to the analytical solution of the semi-infinite medium limit (*Q* → ∞). The shaded area between the two curves represents the range of concentrations expected for finite flow rates *Q*. When *Q* is finite, dilution through the top of the culture is difficult to define and solve numerically (and precludes coupling with a suitable growth model). However, if we assume that the cell culture is diluted uniformly throughout, the domain grows uniformly with time and the problem becomes tractable numerically [19,20]. The numerical solutions for this case are also shown in Figure 3. When *Q* = 0 the solution again reduces to the static culture limit. As *Q* increases, the concentration of TGF-β exposed to the cells decreases. As expected, uniform dilution outperforms dilution through the top because the cells are located at the bottom of the culture. However, for *Q* ≿1 the effect of increasing *Q* becomes negligible. This is consistent with the experimental results of Csaszar and colleagues [8], who observed minimal change in the expansion of HSC populations for *Q* ≿1 ml/day in a 12 ml culture bag. For all values of *Q* the concentration of TGF-β exposed to the cells varies as *ϕ*_cell_ ∝ *t*^1/2^ as *t* → ∞.

The uniform dilution assumption can be considered to give a best-case prediction for practical dilution experiments where medium is added to the top or to the side of the culture. Notably, the effect of diffusion causes the inhibitory signal concentration to increase with time, whereas the instantaneous redistribution assumption predicts that the cell exposure concentration becomes constant, and the value it predicts is far below the value predicted when diffusive effects are considered (Figure 3). Hence, assuming instantaneous redistribution of inhibitory signals throughout the cell culture leads to an underprediction of the concentration of inhibitory proteins exposed to the cells and therefore an overprediction of the effectiveness of a fed-batch culture.

### When spatial gradients of concentration are taken into account, perfusion is a more effective means of reducing exposure of cells to paracrine inhibitory signalling than dilution

**Figure 4:**
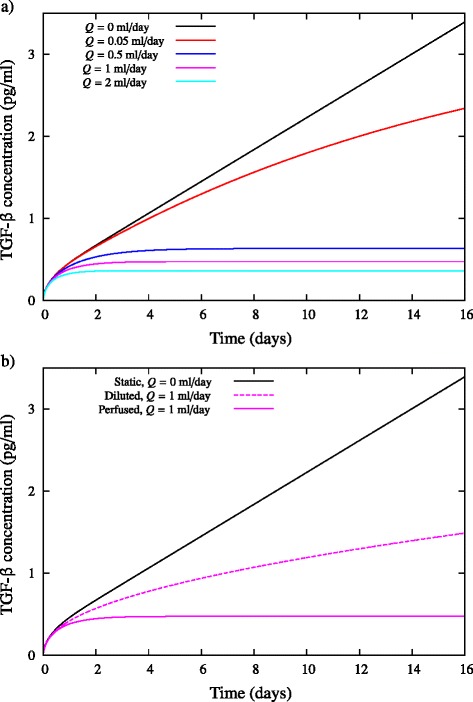
Comparison of diluted and perfused cultures when the effects of diffusion are considered. **(a)** Effect of flow rate *Q* on the average concentration of transforming growth factor beta (TGF-β) at the bottom of a perfused 24-well-plate well of initial media height *h*_0_ = 5 mm (corresponding to 1 ml culture). **(b)** Comparison of fed-batch and perfusion strategies with *Q* = 1 ml/day. The diffusivity is *D* = 6.5 × 10^−11^ m^2^/second, the cell population is assumed constant at *X* = 10^5^, and the secretion rate is *r*_*ϕ*_ = 1.94 × 10^−6^ pg/cell/day. Solid black line is the limiting static case for both strategies with no dilution/perfusion (*Q* = 0 ml/day). The perfused cultures are governed by Equations (5), (12) and (13) in Additional file 1, and the fed-batch cultures by Equations (10) and (11) in Additional file 1.

To determine the effect of perfusion on the concentration of inhibitory signals within a cell culture, a 24-well-plate well of 1 ml volume is assumed to have an inlet and outlet pipe each of radius 1 mm, located halfway up the well. As before, the cell number is fixed at 10^5^ cells and the secretion rate at *r*_*ϕ*_ = 1.94 × 10^−6^ pg/cell/day. The effect of flow rate *Q* on the concentration of inhibitory signals exposed to the cells in the well is shown in Figure 4a. Again, the limiting case for a perfusion flow rate of *Q =* 0 ml/day is the static culture. As *Q* increases, the average concentration of TGF-β exposed to the cells decreases markedly. For perfusion rates *Q* ≿0.5 ml/day, the concentration at the bottom of the well becomes independent of time after 2 to 4 days of culture. To compare against the fed-batch approach, Figure 4b shows the average concentration of TGF-β exposed to the cells for a dilution rate and perfusion rate of *Q* = 1 ml/day. The perfused culture experiences a significantly reduced concentration of inhibitory signal relative to the diluted culture. This is in direct contrast to the infinite-diffusion model of Csaszar and colleagues [8]. Thus, when spatial gradients of concentration are taken into account, perfusion is a far more effective means of reducing exposure of cells to paracrine inhibitory signalling.

**Figure 5:**
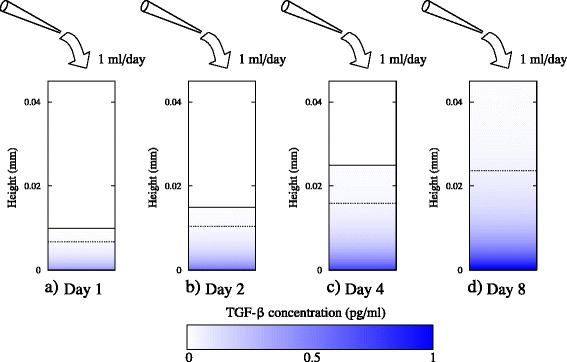
Spatial variation of transforming growth factor beta (TGF-β) concentration within a fed-batch culture. The numerical solutions of Equations (10) and (11) in Additional file 1 are shown for a 24-well-plate well of initial media height *h*_0_ = 5 mm (corresponding to 1 ml culture) for **(a)** 1 day, **(b)** 2 days, **(c)** 4 days, and **(d)** 8 days. The diffusivity is *D* = 6.5 × 10^−11^ m^2^/second, the cell population is assumed constant at *X* = 10^5^, and the secretion rate is *r*_*ϕ*_ = 1.94 × 10^−6^ pg/cell/day. Solid black line indicates the free surface of the cell culture medium, and dashed line indicates the height at which the concentration is 5% of the concentration at the bottom of the culture.

**Figure 6:**
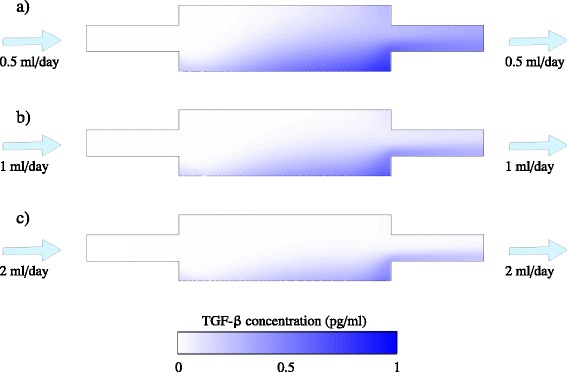
Spatial variation of transforming growth factor beta (TGF-β) concentration within a perfused culture. The numerical solutions of Equations (5), (12) and (13) in Additional file 1 are shown for a 24-well-plate well of media height *h*_0_ = 5 mm (corresponding to 1 ml culture) at day 8, for **(a)** *Q* = 0.5 ml/day, **(b)** *Q* = 1 ml/day and **(c)** *Q* = 2 ml/day. The diffusivity is *D* = 6.5 × 10^−11^ m^2^/second, the cell population is assumed constant at *X* = 10^5^, and the secretion rate is *r*_*ϕ*_ = 1.94 × 10^−6^ pg/cell/day. Contours of TGF-β concentration are shown at the vertical mid-plane of the well.

**Figure 7:**
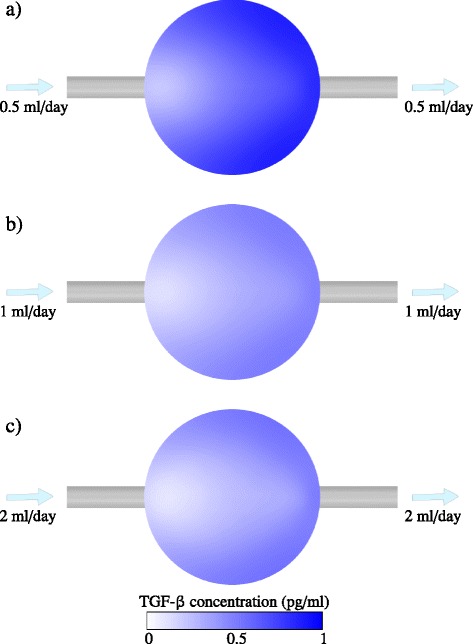
Spatial variation of transforming growth factor beta (TGF-β) concentration at the bottom of a perfused culture. The numerical solutions of Equations (5), (12) and (13) in Additional file 1 are shown for a 24-well-plate well of media height *h*_0_ = 5 mm (corresponding to 1 ml culture) at day 8, for **(a)** *Q* = 0.5 ml/day, **(b)** *Q* = 1 ml/day and **(c)** *Q* = 2 ml/day. The diffusivity is *D* = 6.5 × 10^−11^ m^2^/second, the cell population is assumed constant at *X* = 10^5^, and the secretion rate is *r*_*ϕ*_ = 1.94 × 10^−6^ pg/cell/day. Contours of TGF-β concentration are shown at the bottom surface of the well.

To elucidate why perfusion is more effective than dilution, Figures 5, 6 and 7 compare the spatial variation of TGF-β within the fed-batch and perfused cultures. It is clear that the effect of applying a shear flow across the cell culture is appreciably better than straight dilution. In the perfused culture, the shear flow disperses the concentration distribution in the direction of the flow, significantly diminishing the concentration of TGF-β to which cells are exposed. Further, the presence of shear flow increases the gradient of concentration normal to the bottom of the well, enhancing the diffusion of inhibitory signal away from the cells. This effect is analogous to Taylor dispersion, whereby the application of shear flow to a concentration profile increases the effective diffusivity of the solute [21,22]. Because the flow acts parallel to the surface of the well, an inhomogeneous distribution of inhibitory signal is present on the surface (Figures 6 and 7). However, the maximum concentration present on the surface of the well in the perfusion bioreactor is still much less than in a fed-batch culture.

### The optimal inhibition strategy depends upon the diffusive time scale, measured by the Peclet number

We demonstrate here that the relative importance of diffusion to the media manipulation of any particular mitigation strategy is defined by the Peclet number:

5 $$ \mathrm{P}\mathrm{e} = \frac{\tau_d}{\tau_s}=\frac{L^2}{D{\tau}_s} $$

Here *τ*_*d*_ is the diffusive time scale, *τ*_*s*_ is the media manipulation time scale and *L* is the characteristic length scale, which here is the media height *h*. The media manipulation time scale is dependent on the mitigation strategy being considered. For fed-batch and perfused cultures, the media manipulation time scale is *τ*_*s*_ = *V*_0_/*Q*. For mixed cultures, the media manipulation time scale is *τ*_*s*_ = 1/*f*_mix_. For the media-exchange strategy, where *αV*_0_ ml is exchanged every 1/*f*_ME_ days, the media-manipulation time scale is *τ*_*s*_ = 1/*αf*_mix_*.*

The limit of Peclet number Pe = 0 corresponds to the instantaneous redistribution assumption. To allow direct comparison of the relative effectiveness of each mitigation strategy, the media manipulation time scale is chosen as *τ*_*s*_ = 1 day. Thus, the flow rate of the fed-batch and perfused cultures is *Q* = *V*_0_ ml/day, and the mixing frequency is *f*_ME_ = 1/day. Two different media-exchange strategies with *τ*_*s*_ = 1 day are considered: full media exchange daily (*α* = 1, *f*_ME_ = 1/day); and half media exchange every 12 hours (*α* = 0.5, *f*_ME_ = 2/day). Four different cell cultures are considered: a 12 ml culture bag of initial volume 1 ml, and a well with three different initial volumes of 0.2, 1, and 5 ml. These correspond to initial media heights of 0.33, 1, and 5 mm respectively.

**Figure 8:**
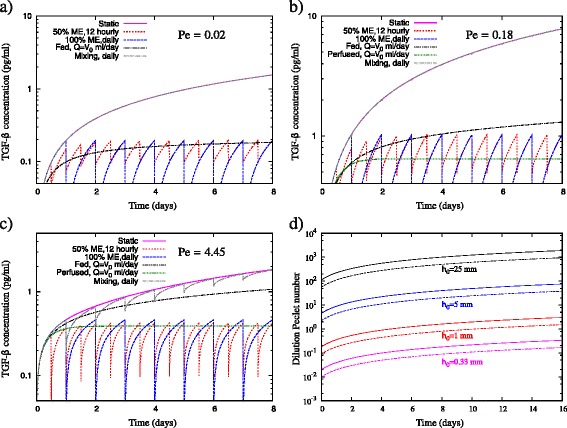
The optimal inhibition strategy depends upon the diffusive time scale, measured by the Peclet number. **(a)** A 12 ml culture bag of initial media height *h*_0_ = 0.33 mm (corresponding to 1 ml culture), **(b)** a 24-well-plate well of initial media height *h*_0_ = 1 mm (corresponding to 0.2 ml culture) and **(c)** a 24-well-plate well of initial media height *h*_0_ = 5 mm (corresponding to 1 ml culture). The diffusivity is *D* = 6.5 × 10^−11^ m^2^/second, the cell population is assumed constant at *X* = 10^5^, and the secretion rate is *r*_*ϕ*_ = 1.94 × 10^−6^ pg/cell/day. **(d)** Change of dilution Peclet number with time for various initial media heights in a 24-well-plate well. Dashed lines represent the dilution Peclet number for an inhibitory signal of diffusivity 2*D*, and solid lines represent the dilution Peclet number for an inhibitory signal of diffusivity *D*, where *D* = 6.5 × 10^−11^ m^2^/second.

For the 1 ml culture in the bag, the Peclet number is 0.02 – indicating that the diffusive time scale is much faster than the media manipulation time scale, and as a consequence the inhibitory signal is able to diffuse throughout the cell culture. The effect of the different mitigation strategies for this case is shown in Figure 8a. At this small Peclet number the effect of each strategy is almost identical to the instantaneous redistribution model shown in Figure 2. As a consequence, mixing has no effect on the mitigation of inhibitory signalling due to the uniform distribution of protein concentration throughout the cell culture. Perfusion is not modelled for this case because it is not a practical strategy for loosely adherent cells in a culture bag; there is no barrier to prevent cells from exiting the device.

The 0.2 ml culture in the 24-well-plate well gives Pe = 0.18, and thus the effect of mixing is still negligible. The fed-batch strategy is more effective at mitigating inhibitory signalling than media exchange for the first 4 days of this culture, but is less effective for longer culture times. For the first day of cell culture, the fed-batch strategy outperforms perfusion, but is much less effective thereafter. Increasing the culture volume to 1 ml in the well also increases the Peclet number to 4.45, indicating that the media manipulation time scale is now faster than the diffusive time scale. As a consequence, mixing becomes a feasible mitigation strategy, because the inhibitory protein only has time to partially diffuse throughout the cell culture before mixing occurs again. For this culture scenario, perfusion and media exchange are the most effective strategies, both clearly outperforming dilution. The dilution strategy is only marginally effective at mitigating inhibitory signalling for this media height because the secreted protein is not able to diffuse away from the cells over the life of the culture.

### The act of dilution increases the Peclet number over time, negating the influence of dilution as a mitigation strategy

For fed-batch cultures, the height of the cell culture increases with time. Thus, the diffusive time-scale increases with time as *τ*_*d*_ ∝ *h*(*t*)^2^, and the dilution media-mitigation time scale increases with time as *τ*_dilute_ ∝ *h*(*t*). The dilution Peclet number Pe_d_ is therefore a function of time and is given by:

6 $$ \mathrm{P}{\mathrm{e}}_{\mathrm{d}} = \frac{{h_0}^2Q\left(1+\frac{Q}{V_0}t\right)}{D{V}_0} $$

The increase in dilution Peclet number is depicted in Figure 8d for various initial media heights. The relationship between the dilution Peclet number and the initial dilution Peclet number (or alternatively the Peclet number for other mitigation strategies with the same media-manipulation time scale) can be shown to be:

7 $$ \frac{\mathrm{P}{\mathrm{e}}_{\mathrm{d}}}{\mathrm{P}{\mathrm{e}}_0} = \left(1+\frac{Q}{V_0}t\right) $$

The diffusive time scale becomes slower in comparison with the dilution time scale over the cell culture period, indicating that the effect of dilution is diminished. This is shown clearly in Figure 5, where the positions of the free surface and the diffusive front are marked in the cell culture. Here the diffusive front is defined as the height in the cell culture where the concentration is 5% of the value at the bottom. After 1 day of culture, there is medium present in the well with very low concentrations of TGF-β relative to the concentration at the bottom of the well. The diffusive time scale at this point in time is approximately nine times greater than the dilution time scale (Pe = 8.9), and there has not been sufficient time for the protein to diffuse uniformly throughout the entire culture. Thus, there are already regions within the cell culture medium with very low concentrations of inhibitory protein. This represents a sink for the inhibitory proteins to diffuse into, and as such there is no benefit in addition of more media by dilution. This is clearly evident in Figure 5: as the cell culture period increases so does the Peclet number, and the position of the diffusive front moves further below the position of the free surface, indicating that there is medium already available within the cell culture for the inhibitory signal to diffuse into. At day 2 the Peclet number is 13.4, and at day 8 the Peclet number of the culture is approximately 40.

**Figure 9:**
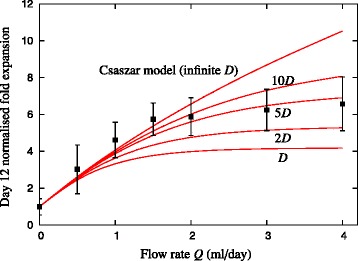
Comparison of growth model predictions with experimental data. Effect of diffusivity on the growth model predictions of fed-batch cultures in a 12 ml culture bag of initial media height *h*_0_ = 0.33 mm (corresponding to 1 ml culture). Here the diffusivity *D* = 6.5 × 10^−11^ m^2^/second, and the experimental points are taken from Csaszar and colleagues [8]. The Csaszar model is defined by Equations (14) to (20) in Additional file 1. For finite diffusivity *D*, the system is governed by Equations (14) to (18), (24) and (25) in Additional file 1. The initial conditions for the growth model are defined in Equation (23) in Additional file 1, and the parameters are presented in Table 2.

To determine the effect of finite diffusivity and also flow rate on cell growth in a fed-batch culture, Figure 9 shows the growth model predictions of total cell expansion in a 12 ml culture bag at day 12 as a function of flow rate for various values of diffusivity. The experimental results of Csaszar and colleagues are also shown [8]. The experimental results show clearly that the effect of increasing the flow rate *Q* on the cell expansion is negligible for *Q* ≿1 ml/day. This result is consistent with the numerical predictions when finite diffusivity is taken into account. The dilution Peclet number (Equation (6)) increases linearly with time, and varies with flow rate as Pe ~ *Q*^2^. Thus, as the flow rate increases, the effect of the resulting increased dilution is negligible. This saturation of cell expansion with flow rate is not captured with the original growth model formulated assuming instantaneous redistribution (that is, *D* → ∞). However, when finite diffusivity is considered, the model is able to capture this experimentally observed effect.

### Cell growth within a perfused culture increases with increasing flow rate, and perfusion is able to sustain expansion at higher cell seeding densities

**Figure 10:**
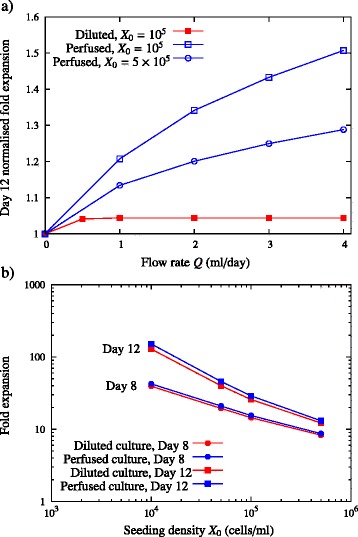
Growth model predictions for perfused and fed-batch cultures of different cell seeding densities *X*_0_ (cells/ml) in a 24-well-plate well of media height *h*_0_ = 5 mm (corresponding to 1 ml culture). **(a)** Normalised fold expansion after day 12 as a function of flow rate *Q*, and **(b)** fold expansion after day 8 and day 12 as a function of initial seeding density *X*_0_. The fed-batch growth model is defined by Equations (14) to (18), (24) and (25) in Additional file 1, and the perfusion growth model by equations (14) to (18) and (25) to (27) in Additional file 1. The diffusivity *D* = 6.5 × 10^−11^ m^2^/second, the initial conditions for the growth model are defined in Equation (30), and the parameters are presented in Table 2.

The effect of flow rate on cell growth is shown in Figure 10a for both perfused and fed-batch cultures. Unlike the fed-batch culture, no saturation with flow rate is observed over the range of flow rates modelled. Instead, the cell growth within the perfused culture increases with increasing flow rate, consistent with the predictions based on the assumption of fixed cell density. Even at high initial seeding densities, *X*_*d*_ ≿5 × 10^5^ cells/ml, perfusion clearly outperforms dilution, and its effectiveness is enhanced with increasing flow rate. The effect of initial cell seeding volume density is qualified in Figure 10b. For all seeding densities modelled, the perfused culture outperforms the diluted cultures. This effect is most pronounced at low initial seeding densities.

### Mixing and media exchange strategies become more effective as the Peclet number increases

**Figure 11:**
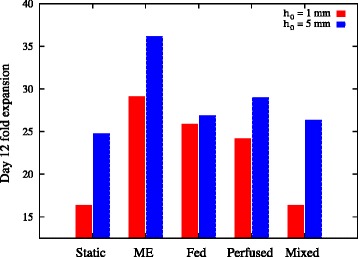
Growth model predictions of population expansion after 12 days of culture for different mitigation strategies. Model predictions are shown for 24-well-plate wells of initial media height *h*_0_ = 1 mm (red bars) and *h*_0_ = 5 mm (blue bars), corresponding to 0.2 ml and 0.5 ml culture volumes respectively. The fed-batch growth model is defined by Equations (14) to (18), (24) and (25) in Additional file 1, and the perfusion growth model by Equations (14) to (18) and (25) to (27) in Additional file 1. The diffusivity *D* = 6.5 × 10^−11^ m^2^/second, the initial conditions for the growth model are defined in Equation (30), and the parameters are presented in Table 2.

Figure 11 shows the predicted cell expansion after 12 days of culture for the mitigation strategies considered in 24-well-plate wells of initial media heights *h*_0_ = 1 mm and *h*_0_ = 5 mm (corresponding to Peclet numbers of Pe = 0.18 and Pe = 4.45 respectively). For both media heights considered, media exchange presents as the best strategy because it removes all inhibitory signals from the culture. Perfusion is less effective than dilution for the culture with initial media height of 1 mm, and mixing for this culture height gives no advantage over the cell expansion in a static culture. For the media height of 5 mm (and therefore higher Peclet number), there is only a marginal increase in effectiveness of the dilution strategy, consistent with the earlier observation that there are already regions of media with very low inhibitory concentration present in the cell culture. In contrast, there is a significant increase in the effectiveness of the perfusion strategy as the media height increases. At the media height of 5 mm, the mixing strategy offers a significant improvement over the static culture, and is almost as effective as the diluted culture.

## Discussion

This study has presented numerical models of mixed, media-exchange, fed-batch and perfused cultures that take into account the finite mass of secreted paracrine signals, allowing spatial concentration gradients to form within cell cultures. A growth model is also developed that takes into account the finite mass of secreted factors. The models presented here show that the optimal mitigation strategy is dependent on the Peclet number, which measures the size of the diffusive time scale relative to the media-manipulation time scale. When the Peclet number is very small, the inhibitory protein redistributes quickly throughout the cell culture, and the fed-batch approach is a useful strategy. However, we have shown that there is limited scope for tunable feedback control in fed-batch cultures unless the Peclet number remains very small over the life of the cell culture. Even for low initial Peclet numbers (Pe_0_ ≿0.2), the effectiveness of dilution diminishes over the cell culture period. This is because the action of dilution causes the cell culture volume to continually increase, and consequently the Peclet number also increases with time. This increase in Peclet number over time diminishes the effectiveness of dilution as a means for reducing the concentration of inhibitory proteins where cells are actually growing.

As the Peclet number increases, the size of the diffusive time scale increases relative to the dilution time scale, and there are regions of media with very low inhibitory concentration already present in the cell culture. Hence, the addition of more fresh media to the culture has negligible effect. This prediction is consistent with previous experimental data on fed-batch cultures. Further, the tunability of fed-batch cultures is also restricted by the dilution Peclet number increasing with flow rate as Pe ∼ *Q*^2^, whereas for other mitigation strategies with constant cell culture volume the Peclet number is only linearly proportional to the flow rate. For moderate to high Peclet numbers, the numerical models predict that media exchange is the best strategy, but as a strategy it is labour intensive, especially if the frequency of exchange is shorter than 1 day. Further, media exchange is too difficult to implement in large-scale culture systems. At moderate to high Peclet numbers, mixing represents the most economical method, with significant mitigation predicted without need for extra media to be added to the cell culture.

It has been shown here that perfusion-based systems are more effective at mitigating the effects of inhibitory signalling in HSC cultures than dilution-based approaches. Other strategies where cell culture volume is kept constant are more amenable to tunability than dilution, especially perfusion. Increasing the flow rate increases the effectiveness of the perfusion strategy, and thus has significant scope as an automated, tunable strategy for optimising cell expansion. Furthermore, the perfusion culture scenarios considered here are not optimised. Significant improvement in efficiency may be achieved through enhancing the design of the perfusion device and its operating conditions. There are extensive reports on the fluid flow and nutrient distribution within perfusion bioreactors [23-28]. Using the growth model presented here to predict the distribution of inhibitory factors and the subsequent effect on cell expansion in these devices will allow efficient assessment of design and operating conditions to optimise expansion whilst eliminating the need for expensive and time-consuming prototype iterations.

The growth model as it stands will underestimate cell expansion in perfused cultures because perfusion, shown here to effectively lower the concentration of endogenous inhibitory factors, will also act to lower the concentration of endogenous stimulatory factors and hence also lower the predicted cell expansion. However, the growth model does not take into account exogenous stimulatory factors that are already present at very high concentrations in the supplied media. The effect of perfusion on the stimulatory factor concentration will thus be minimised, as fresh stimulatory factors will be brought in to replace those swept away. Kirouac presented a variation of the original model that did not include the positive proliferation feedback factor *ϕ*_3_, showing that it was a slightly better fit to the experimental data [29]. However, both stimulatory factors were retained in the presented model to fully capture the effect of positive feedback loops observed *in vivo* [30]. There is thus a need to reformulate the growth model to remove the influence of positive feedback loops, and to account for exogenous stimulatory factors already present, in order to fully capture the overall effect of these types of mitigation strategies. There is also a need to reformulate the growth model in terms of cell-to-surface-area ratio, rather than cell seeding concentration. For example, cells in a culture seeded at 10^5^ cells/ml are packed much closer together in a standard tissue culture 24-well-plate well than on the surface of a 12 ml culture bag. Assuming that the cells are of diameter ~15 μm, this corresponds to an initial average distance between cells of ~40 μm in the well, and ~170 μm in the culture bag. Reformulating the growth model to account for the cell-to-surface area ratio will allow more accurate predictions of cell growth, and also capture the effect of heterogeneous inhibitory signal distribution within perfused and diluted cultures.

The growth model used here suggests that there is an upper limit to growth for loosely adherent cells within a specified surface area, independent of the mitigation strategy used. Quantification of this upper limit would require new estimates of the growth model parameters. However, it can be conjectured that once the surface area of a cell culture has been exhausted (that is, the cells become fully confluent), cells will begin to stack one upon another. As a result, strategies such as perfusion and dilution will be unable to mitigate against paracrine signalling for cells not on the uppermost layer. Thus, supplementary strategies such as cell redistribution and mixing of the entire cell culture may become critical at high cell densities to push cell expansion beyond the limit possible with perfusion/dilution alone.

The optimal approach to mitigate inhibitory signalling may consist of changing from one strategy to another during the life of the cell culture depending upon levels of paracrine signalling present, and the Peclet number of the cell culture. For example, dilution could be used initially as it appears to be the most effective strategy in the early stages of cell culture, and then perhaps perfusion, media exchange or mixing could be utilised to further enhance cell expansion. The models described herein provide the basis for a detailed cost analysis, specifically for the expensive growth factors that will be consumed at different amounts depending on the culture manipulation and feeding strategy being deployed. However, more knowledge of specific inhibitory proteins and how they act in combination is required to determine optimal mitigation strategies. This is particularly relevant in cultures where a heterogeneous mix of stem cells and differentiated cells are present. In this case – as often occurs with the asynchronous division and differentiation of HSCs – maturing, differentiated cells can generate a mixture of inhibitory proteins and these may act cooperatively to inhibit nascent primitive haemopoietic progenitors present in culture.

Future work will focus on developing a more generalised growth model, which also takes into account the difference in diffusivities of different inhibitory factors. In particular, a significant improvement would be the inclusion of stochastic modelling of individual factor trajectories, such as the approach developed by Moledina and colleagues [31], giving rise to cell population heterogeneity in the culture.

## Conclusions

Numerical models of HSC cultures that incorporate inhibitory signalling and the effects of finite protein size are presented. The models are applied to determine the effectiveness of various strategies used to mitigate the effects of inhibitory signalling inherent in these cultures. The strategies assessed include mixing, media-exchange, fed-batch and perfusion approaches. The results show that the optimal approach to mitigate inhibitory signalling in HSC cultures is dependent upon the relative sizes of the protein diffusion time scale relative to the time scale of the media manipulation.

### Endnote

^a^Here and throughout the paper the secretion rate is expressed in terms of actual cell number *X*, rather than the normalised cell number $\tilde{X}$ used by Kirouac and colleagues [9].

## Additional files

Additional file 1:
**Presents details of the governing equations and numerical models presented in this study.**
Additional file 2: Figure S1. Showing comparison of the one-dimensional dilution model (which neglects advection) and the three-dimensional dilution model (which includes advection) for constant cell population *X* = 10^5^. Black dots indicate the results of the three-dimensional dilution model, which includes the effects of advection. Top subfigure is a 24-well-plate well of initial media height of 1 mm, and bottom subfigure is a 24 well-plate-well of initial media height 5 mm. The computational fluid dynamics package Fluent (ANSYS, Canonsburg, PA, USA) was used with dynamic meshing to capture the effects of advection in the three-dimensional dilution case. The base mesh used in these simulations was identical to the mesh used in the perfusion cases presented in the paper. Additional file 3: Figure S2. Showing comparison of the growth model predictions for the one-dimensional (1D) dilution model (which neglects advection) and the three-dimensional (3D) dilution model (which includes advection) for a cell culture in a 12 ml culture bag of initial volume 1 ml. The computational fluid dynamics package Fluent (ANSYS, Canonsburg, PA, USA) was used with dynamic meshing to capture the effects of advection in the three-dimensional dilution case. The base mesh used in these simulations was identical to the mesh used in the perfusion cases presented in the paper.

## Abbreviations

HSC: haemopoietic stem cell
ME: media exchange
Pe: Peclet number
SF: secretion factor
TGF-β: transforming growth factor beta
