# Supplementary material for: Predictions for optimal mitigation of paracrine inhibitory signalling in haemopoietic stem cell cultures
Source: Stem Cell Res Ther. 2015 Apr 16;6(1):58. doi: 10.1186/s13287-015-0048-7 (PMC4443622; doi:10.1186/s13287-015-0048-7)
Supplement: Additional file 1: — Presents details of the governing equations and numerical models presented in this study. [file 13287_2015_48_MOESM1_ESM.pdf]

Theoretical Framework

Transport Equations

Static, mixed and media-exchange cultures

The distribution of a secreted protein concentration $\phi(\boldsymbol{x}, t)$ with diffusivity D in a cell culture is governed by the transient diffusion equation

$$\frac{\partial \phi}{\partial t} = D\nabla^2 \phi. \tag{1}$$

The amount of protein secreted by the cells at time t is given by $r_\phi X(t)$, where r_ϕ is the cell secretion rate of ϕ (assumed to be independent of time) and $X(t)$ is the number of secreting cells. Assuming that the cells are located at the bottom of the cell culture of surface area A, the flux of ϕ secreted by the cells is

$$q_\phi(t) = r_\phi \frac{X}{A}. \tag{2}$$

Under the assumption that the cell to surface area ratio $X(t)/A$ is uniform, equation (1) reduces to the one-dimensional equation

$$\frac{\partial \phi}{\partial t} = D\frac{\partial^2 \phi}{\partial y^2}, \tag{3}$$

with boundary conditions

$$\frac{d\phi}{dy}(0,t) = -\frac{q_\phi}{D}, \quad \frac{d\phi}{dy}(h,t) = 0. \tag{4}$$

The first boundary condition represents the flux of ϕ secreted by the cells at $y = 0$, and the second represents zero flux of ϕ through the top surface of the cell culture. Here h is the height of the free surface in the cell culture. It is assumed that at the start of the cell culture period that the initial secreted protein concentration is zero.

To model the effect of mixing at specified times in the cell culture period, the distribution of inhibitory concentration ϕ throughout the cell culture was set to be uniform and equal to the average concentration at that particular instant. For full media exchange at specified times, ϕ was set to zero. For half media exchange, ϕ was set to be uniform and equal to half the average concentration at that particular instant.

Fed-batch cultures

In fed-batch cultures the spatial and temporal distribution of ϕ is governed by the transient advection-diffusion equation (1)

$$\frac{\partial\phi}{\partial t} + \nabla\cdot(\boldsymbol{u}\phi) = D\nabla^2\phi. \tag{5}$$

where the height $h(t)$ of the free surface varies with time under the action of dilution. Under the assumption that the surface cell density $X(t)/A$ is uniform, equation (5) reduces to

$$\frac{\partial\phi}{\partial t} + \frac{\partial(v\phi)}{\partial \mathrm{y}} = D\frac{\partial^2\phi}{\partial y^2}. \tag{6}$$

Under the assumption that the culture is continuously fed with uniform volume flow rate Q, any point $y(t)$ in the domain can be represented by the Lagrangian description

$$y = y_0 r(t), \;\; r(t) = \left(1 + \frac{Q}{V_0} t\right), \tag{7}$$

where y_0 is the initial position marker, and $r(t)$ is the growth-rate of the domain. Hence, the velocity v due to the action of continuous uniform dilution is

$$v = \frac{\partial y}{\partial t} = y_0 \dot{r} = y \frac{\dot{r}}{r}. \tag{8}$$

Thus, equation (6) becomes

$$\frac{\partial \phi}{\partial t} + \frac{\dot{r}}{r}\left(y \frac{\partial \phi}{\partial \mathrm{y}} + \phi\right) = D \frac{\partial^2 \phi}{\partial y^2}. \tag{9}$$

Upon transformation to the unit interval $(y, t) \rightarrow (y^{'}, t^{'}) = (y/h(t)\,, t)$, equation (9) becomes

$$\frac{\partial \phi}{\partial t'} = \frac{D}{h(t')^2} \frac{\partial^2 \phi}{\partial y'^2} - \frac{\dot{r}}{r} \phi. \tag{10}$$

Here $h(t') = h(t) = \; h_0 r(t)$ is the height of the cell culture medium at time t.

In the same way, the boundary conditions given in equation (4) become

$$\frac{d\phi}{dy'}(0, t') = -h(t') \frac{q_\phi}{D}, \;\; \frac{d\phi}{dy'}(1, t') = 0. \tag{11}$$

Perfused cultures

The distribution of $\phi(\boldsymbol{x}, t)$ within a perfused cell culture is governed by the transient advection-diffusion equation (Equation 5), whereby the fluid velocity $\boldsymbol{u}$ is defined by the

solution of the Navier-Stokes equation in conjunction with the continuity equation, defined respectively as

$$\rho\left(\frac{\partial \boldsymbol{u}}{\partial t}+\boldsymbol{u}\cdot\nabla\boldsymbol{u}\right)=-\nabla p+\mu\nabla^2\boldsymbol{u}, \tag{12}$$

$$\text{and } \nabla\cdot\boldsymbol{u}=0, \tag{13}$$

where ρ is the medium density, μ is the medium viscosity, and p is the pressure. The fluid velocity within a cell culture is dependent upon the geometry of the culture chamber and Equation 5 cannot be simplified to one dimension. The boundary conditions for the inhibitory protein ϕ in this case are the same as Equation 4, with prescribed flux at the bottom of the bioreactor where the cells reside, and zero diffusive flux at all other boundaries.

Growth model

The growth model developed here can be used to provide predictions of the effect of flow rate and cell seeding density on cell growth in cell cultures under various mitigation strategies. The parameters defined by Kirouac et al (2) were chosen in their study to fit the experimental data of Madlambayan et al (3) for static cultures (with media exchange), under the assumption of instantaneous redistribution. The values of these 16 parameters are given in Table 2. The growth model introduces four new parameters, namely the diffusivity D_i of each of the four secreted factors, in order to capture the spatial concentration gradients present in the cell cultures. For quantitative predictions of growth, it would be necessary to recalibrate the 16 original parameters, and the 4 new parameters, of the growth model using new experimental data. Further, the model is expressed in terms of cell volume density, and would need to be recast in terms of cell surface density to be applicable to a wide range of devices. However, the model as it stands can be used to qualitatively assess the expansion of cell cultures undergoing perfusion relative to fed-batch cell cultures. In this respect, the

model predicts the growth of a synthetic cell population with both stimulatory and inhibitory signalling.

A growth model specific to HSC culture incorporating the effects of paracrine signalling was first developed by Kirouac et al. (2, 4). The model divides the haemopoietic hierarchy into twenty cellular compartments $\tilde{X}_i$ (defined in Figure 1 of Kirouac et al (2)). The growth rates of each cell type are defined by the equations

$$\frac{d\tilde{X}_i}{dt} = 2(1 - f_{i-1})u_{i-1}\tilde{X}_{i-1} + (2f_i - 1)u_i\tilde{X}_i \text{ for } i = 1,2, \dots ,20. \tag{14}$$

Here $\tilde{X}_i$ is the (normalised) number of cells in each compartment[1]. The proliferation rates u_i and the self-renewal rates f_i appearing in equation (14) are defined respectively as

$$u_i = u_{max}\exp\left(\frac{-\left(i - n_{\mathrm{max})}\right)^2}{2{D_{gr}}^2}\right)\left(\frac{t^{k_t}}{{\tau_D}^{k_t} + t^{k_t}}\right)\left(\frac{1 + {\phi_3}^{k_3}}{1 + {\phi_1}^{k_1} + {\phi_3}^{k_3}}\right) \tag{15}$$

$$\text{for } i = 1,2, \dots ,12,$$

$$u_i = u_+ \left(\frac{t^{k_t}}{{\tau_D}^{k_t} + t^{k_t}}\right)\left(\frac{1}{1 + {\phi_1}^{k_1}}\right) \text{ for } i = 13,14, \dots ,20, \tag{16}$$

$$f_i = f_{max}\exp\left(\frac{-(i-1)^2}{2{D_{sr}}^2}\right)\left(\frac{1 + {\phi_4}^{k_4}}{1 + {\phi_2}^{k_2} + {\phi_4}^{k_4}}\right) \text{ for } i = 1,2, \dots ,8, \text{ and} \tag{17}$$

$$f_i = f_{max}\exp\left(\frac{-i^2}{2{D_{sr}}^2}\right)\left(\frac{1 + {\phi_4}^{k_4}}{1 + {\phi_2}^{k_2} + {\phi_4}^{k_4}}\right) \text{ for } i = 9,10, \dots ,20. \tag{18}$$

[1] The original growth model developed by (Kirouac et al (2)) normalised the number of cells in each compartment by the factor $8191/10^5$. In this study the symbol $\tilde{X}$ represents the normalised cell number of the original growth model, and the symbol X refers to the actual cell number.

The parameters appearing in equations (15)-(18) are defined in Table 2. Here, ϕ_k are the four secreted factors, of which two are inhibitory and two are stimulatory.

Instantaneous redistribution assumption

Assuming instantaneous redistribution throughout the cell culture, the equations describing the growth rates of the four secreted factors in a fed-batch culture are defined as

$$\frac{d\phi_k}{dt} = \frac{1}{V_0 + Qt}\left[r_k\left(\sum\nolimits_{i=14}^{20} \tilde{X}_i\right) - Q\phi_k\right] \text{ for } k = 1,3,4 \tag{19}$$

$$\frac{d\phi_2}{dt} = \frac{1}{V_0 + Qt}\left[r_2\left(\frac{{\phi_1}^{k_s}}{{L_s}^{k_s} + {\phi_1}^{k_s}}\right)\left(\sum_{i=1}^{13} \tilde{X}_i\right) - Q\phi_2\right] \text{for } k = 2 \tag{20}$$

Alternatively, for a perfused culture, the growth-rate equations for ϕ_k are

$$\frac{d\phi_k}{dt} = \frac{1}{V_0}\left[r_k\left(\sum\nolimits_{i=14}^{20} \tilde{X}_i\right) - Q\phi_k\right] \text{ for } k = 1,3,4 \tag{21}$$

$$\frac{d\phi_2}{dt} = \frac{1}{V_0}\left[r_2\left(\frac{{\phi_1}^{k_s}}{{L_s}^{k_s} + {\phi_1}^{k_s}}\right)\left(\sum_{i=1}^{13} \tilde{X}_i\right) - Q\phi_2\right] \text{ for } k = 2 \tag{22}$$

The initial conditions for the growth model are (4)

$$\tilde{X}_i^{\,0} = \begin{cases} \frac{X_d}{10^5} 2^{(i-1)} & \text{for } i = 1,2,\dots,13 \\ 0 & \text{for } i = 14,15,\dots,20. \end{cases} \tag{23}$$

Here X_d is the initial cell seeding density (cells/ml). The model was initially applied to static cultures ($Q = 0$ ml/day), and extended to perfused and fed-batch cultures under the assumption of instantaneous redistribution of secreted proteins (5). Thus, to capture the effects of protein size and hence diffusivity on cell population growth, the equations

governing the evolution equations of the four secreted factors in the model of Kirouac et al. (equations 19 & 20) need to be modified to account for the effects of finite diffusion.

Finite diffusion: fed-batch cultures

Following the formulation described previously, the evolution equations for the four secreted factors can be written as

$$\frac{\partial \phi_k}{\partial t'} = \frac{D_k}{h(t')^2}\frac{\partial^2 \phi_k}{\partial y'^2} - \frac{\dot{r}}{r}\phi_k \text{ for } k = 1,2,3,4, \tag{24}$$

with boundary conditions for each factor given by Equation 11, and the fluxes q_k defined as

$$q_k = \begin{cases} \dfrac{r_k}{A}\displaystyle\sum_{i=14}^{20}\tilde{X}_i \text{ for } k = 1,3,4 \\ \dfrac{r_k}{A}\left(\dfrac{{\phi_1}^{k_s}}{{L_s}^{k_s} + {\phi_1}^{k_s}}\right)\displaystyle\sum_{i=1}^{13}\tilde{X}_i \text{ for } k = 2 \end{cases} \tag{25}$$

Note that when the flow-rate $Q = 0$, then $\dot{r}/r = 0$ and $\mathrm{h}(t) = h_0$, corresponding to a static cell culture with no dilution.

Finite diffusion: perfusion cultures

Following the formulation presented previously, the evolution equations for the four secreted factors are

$$\frac{\partial \phi_k}{\partial t} = D_k \nabla^2 \phi_k - \boldsymbol{u} \cdot \boldsymbol{\nabla} \phi_k \text{ for } k = 1,2,3,4 \tag{26}$$

with prescribed fluxes as defined in Equation 25 of ϕ_k at the bottom of the bioreactor where the cells reside, and zero diffusive flux at all other boundaries. The fluid velocity $\boldsymbol{u}$ within the cell culture is found by solving the Navier-Stokes equation and continuity equation.

Unlike fed-batch and static cultures, the values of ϕ_k vary across the bottom of the well when the culture is perfused. Thus, in order to couple the transport equations for ϕ_k with the growth model equations, the average values of ϕ_k on the bottom surface of the well are used. These are defined as

$$\bar{\phi}_k = \frac{1}{A}\int_{bottom} \phi_k \, dA \tag{27}$$

Thus ϕ_k in equations (15)-(18) and (25) is replaced with $\bar{\phi}_k$.

For all cases considered, the diffusivity of each secreted factor is set to be equal to that of TGF-β as a first approximation.

References

1. Crampin EJ, Gaffney Ea, Maini PK. Reaction and diffusion on growing domains: scenarios for robust pattern formation. Bulletin of mathematical biology. 1999;61:1093-120.
2. Kirouac DC, Madlambayan GJ, Yu M, Sykes EA, Ito C, Zandstra PW. Cell-cell interaction networks regulate blood stem and progenitor cell fate. Mol Syst Biol. 2009;5:293.
3. Madlambayan GJ, Rogers I, Kirouac DC, Yamanaka N, Mazurier F, Doedens M, et al. Dynamic changes in cellular and microenvironmental composition can be controlled to elicit in vitro human hematopoietic stem cell expansion. Experimental hematology. 2005;33:1229-39.
4. Kirouac D. Intercellular Feedback in Hematopoiesis. Toronto: University of Toronto; 2009.
5. Csaszar E, Kirouac DC, Yu M, Wang WJ, Qiao WL, Cooke MP, et al. Rapid Expansion of Human Hematopoietic Stem Cells by Automated Control of Inhibitory Feedback Signaling. Cell Stem Cell. 2012;10(2):218-29.
