# Supplementary figures and images for: Predictions for optimal mitigation of paracrine inhibitory signalling in haemopoietic stem cell cultures
Source: Stem Cell Res Ther. 2015 Apr 16;6(1):58. doi: 10.1186/s13287-015-0048-7 (PMC4443622; doi:10.1186/s13287-015-0048-7)

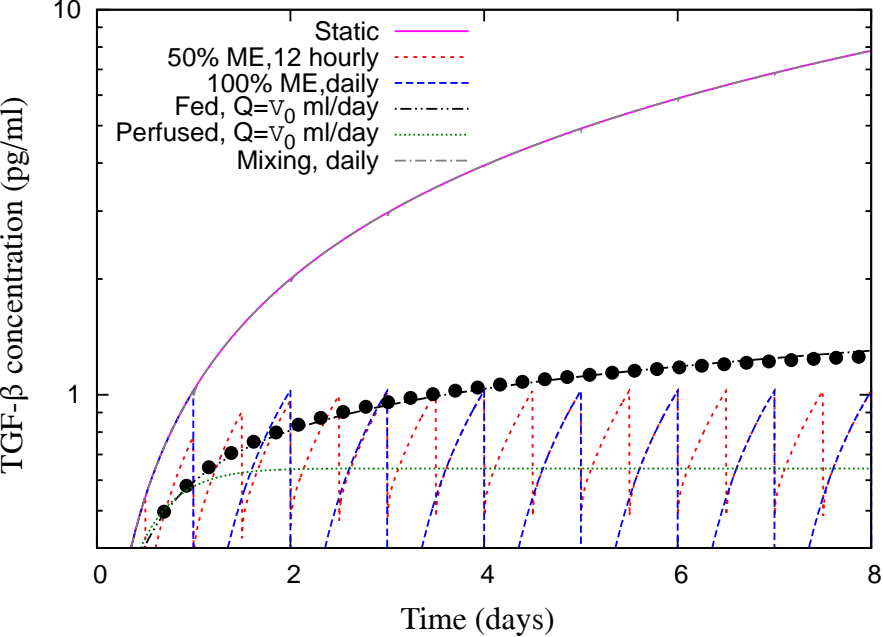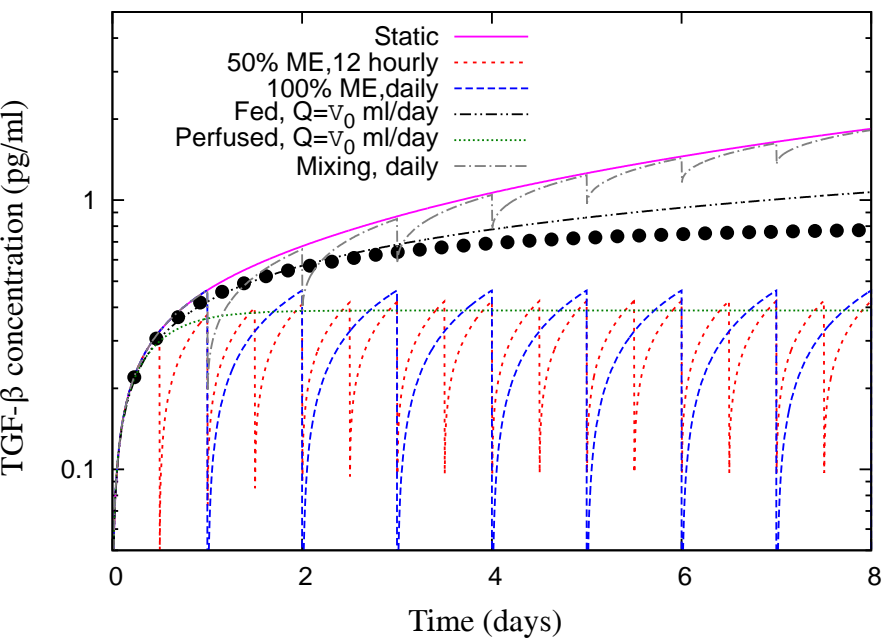

Supplement: Additional file 2: Figure S1. — Showing comparison of the one-dimensional dilution model (which neglects advection) and the three-dimensional dilution model (which includes advection) for constant cell population X = 105. Black dots indicate the results of the three-dimensional dilution model, which includes the effects of advection. Top subfigure is a 24-well-plate well of initial media height of 1 mm, and bottom subfigure is a 24 well-plate-well of initial media height 5 mm. The computational fluid dynamics package Fluent (ANSYS, Canonsburg, PA, USA) was used with dynamic meshing to capture the effects of advection in the three-dimensional dilution case. The base mesh used in these simulations was identical to the mesh used in the perfusion cases presented in the paper. [file 13287_2015_48_MOESM2_ESM.pdf]

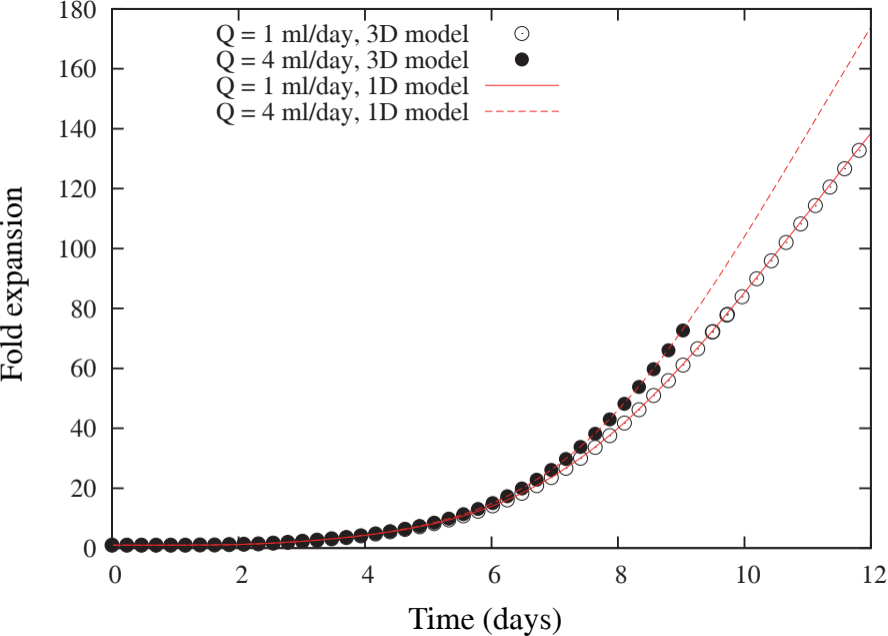

Supplement: Additional file 3: Figure S2. — Showing comparison of the growth model predictions for the one-dimensional (1D) dilution model (which neglects advection) and the three-dimensional (3D) dilution model (which includes advection) for a cell culture in a 12 ml culture bag of initial volume 1 ml. The computational fluid dynamics package Fluent (ANSYS, Canonsburg, PA, USA) was used with dynamic meshing to capture the effects of advection in the three-dimensional dilution case. The base mesh used in these simulations was identical to the mesh used in the perfusion cases presented in the paper. [file 13287_2015_48_MOESM3_ESM.pdf]
